# Evaluation of right adrenal vein anatomy by Dyna computed tomography in patients with primary aldosteronism

**DOI:** 10.1038/srep28305

**Published:** 2016-06-23

**Authors:** Bo-Ching Lee, Chin-Chen Chang, Kao-Lang Liu, Yeun-Chung Chang, Vin-Cent Wu, Kuo-How Huang

**Affiliations:** 1 Department of Medical Imaging, National Taiwan University Hospital and National Taiwan University College of Medicine, Taipei, Taiwan; 2 Department of Internal Medicine, National Taiwan University Hospital and National Taiwan University College of Medicine, Taipei, Taiwan; 3 Department of Urology, National Taiwan University Hospital and National Taiwan University College of Medicine, Taipei, Taiwan

## Abstract

Primary aldosteronism (PA) is the most common cause of secondary hypertension and consists up to 11% of patients with hypertension. Adrenal venous sampling (AVS) is the recommended procedure for diagnosis of PA, but the technique is difficult and the right adrenal vein is especially hard to catheterize. We retrospectively examined the clinically relevant anatomy of the right adrenal vein in a sample of 66 PA patients with technically successful AVS and distinctly-opacified right adrenal veins in Dyna computed tomography (CT). In the majority of cases: the right adrenal veins were catheterized when the catheter tilted posterior and rightward (57/66, 86.4%), the transverse direction of the right adrenal vein from the inferior vena cava (IVC) was posterior and rightward (55/66, 83.3%), and the vertical direction of the right adrenal vein from the IVC was caudal (52/66, 78.8%). This study shows that Dyna CT is able to provide detailed anatomical information to the course and direction of the right adrenal vein.

Primary aldosteronism (PA) is now considered the most common cause of secondary hypertension, with an estimated prevalence of up to 11% in unselected hypertensive patients^1^. Common underlying etiologies include an aldosterone-producing adenoma, unilateral adrenal hyperplasia, and bilateral idiopathic adrenal hyperplasia^2^,^3^,^4^. PA can be divided into unilateral and bilateral subtypes. Surgery is indicated for patients with unilateral hyperaldosteronism, which is able to alleviate or cure hypertension in most patients^5^,^6^. Thus, the lateralization of PA is crucial for the determination of further treatment strategies.

It has been shown that computed tomography (CT) and magnetic resonance imaging (MRI) can be misleading and unreliable for distinguishing unilateral from bilateral PA^7^,^8^,^9^. Adrenal venous sampling (AVS) is considered the gold standard for determining the laterality of PA via the direct sampling of aldosterone secretion. The application of this approach in all PA patients is also recommended by the Endocrine Society^9^. However, AVS is a technically challenging procedure because the right adrenal vein is small and difficult to catheterize. The reported success rates of AVS vary widely, from 55–97%^7^,^10^,^11^,^12^.

A thorough understanding of the right adrenal venous anatomy is essential to proper catheter selection and target-oriented searching for the right adrenal vein. Nevertheless, previous anatomical reports gave little attention to the orientation and orificial location of the right adrenal vein^13^,^14^, which is of paramount importance to AVS procedures. Recently, the presence of Dyna CT and its ability to delineate the vascular structures have increased the success rate of AVS^10^,^15^. To our knowledge, a detailed description of the clinical relevant anatomy of the right adrenal vein incorporating Dyna CT images is lacking. The aim of this study was to determine the location and anatomical variation of the right adrenal vein using Dyna CT during AVS.

## Materials and Methods

**Figure 1:**
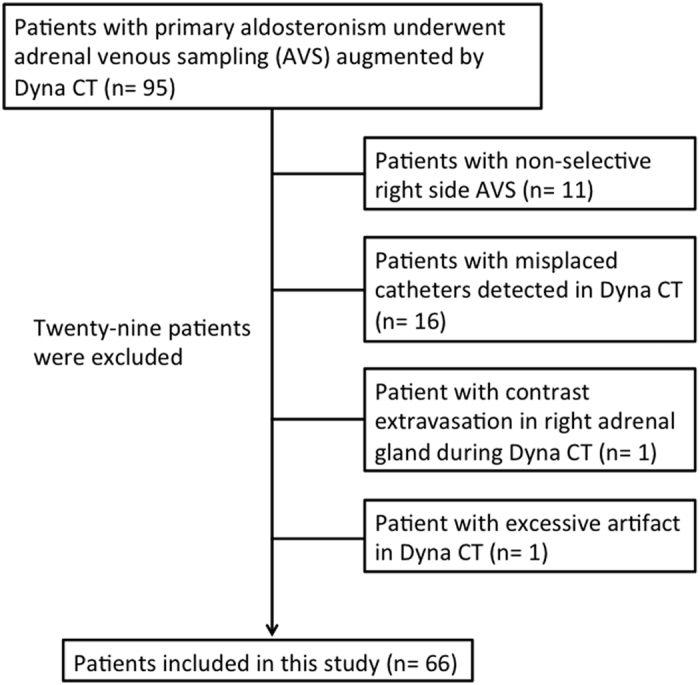
Flow chart of the analysis of Dyna CT images.

The current study was Health Insurance Portability and Accountability Act compliant, and was approved by the institutional review board of the National Taiwan University Hospital with informed consent waived. We retrospectively analyzed the Dyna CT images of 95 consecutive patients who had AVS between April 2012 and December 2014 from the Taiwan Primary Aldosteronism investigator (TAIPAI) group database. Based on predetermined exclusion criteria, 29 patients in this sample were subsequently excluded from further analysis. These exclusion criteria included sampling result of the right AVS that did not meet the selectivity criteria ([cortisol]_right adrenal vein_/[cortisol]_IVC_ >2.0 or [aldosterone]_right adrenal vein_/[aldosterone]_IVC_/>2.0)^16^,^17^ (*n* = 11), misplaced catheters in Dyna CT images (*n* = 16), poor visibility of the right adrenal vein due to contrast extravasation (*n* = 1), and excessive beam-hardening artifact from contrast medium (*n* = 1). Thus, a total of 66 patients who had undergone selective right AVS were retrospectively enrolled in this study (Fig. 1).

### AVS protocol

Informed consent was obtained from all patients before AVS. All AVS was performed between 8:00 AM and 11:00 AM, in order to avoid any bias arising from the aldosterone circadian rhythm^18^. The patients were encouraged to maintain a supine position one hour before the AVS procedures to avoid artificial aldosterone gradient. No adrenocorticotropic hormone (ACTH) stimulation was administered before or during AVS. We adopted several ways to minimize the patient stress during AVS, including pre-procedural explanation to the patient, reassurance by the medical staffs, and real-time echo-guidance femoral vein puncture to lessen discomfort. A 4-French C1 catheter with a side hole (Torcon NB, Cook Medical, Bloomington, U.S.A.) was used in venous sampling of bilateral adrenal veins and the IVC to measure plasma aldosterone and cortisol in most instances. All the procedures were performed by the same radiologist (C.-C.C.), who had 6 years of experience with AVS. IVC was sampled at the beginning of the procedure before adrenal catheterization. The position of the catheter tip before sampling the right adrenal vein was checked via the injection of a small amount of diluted contrast medium (Omnipaque 350, GE healthcare, Carrigtohill, Ireland) and Dyna CT. We routinely discarded the first 10 ml of blood during IVC sampling and the first 5 ml of blood during adrenal sampling to avoid admixture with the intraluminal contrast medium.

### Dyna CT protocol

All Dyna CT procedures were performed using a ceiling mounted angiography system (Zeego Artis, Siemens, Erlangen, Germany). The patient’s arm was placed over their head to reduce radiation exposure during Dyna CT scanning. The isocenter was set in an anteroposterior and lateral position to ensure the presence of the right adrenal vein in the field of view (FOV). Thereafter, a test run was performed to avoid any collision. We diluted the contrast medium with normal saline in a 1:3 ratio to avoid an excessive streak artifact resulting from the injection. The actual Dyna CT was acquired 2 seconds after the start of the injection of contrast medium. The diluted contrast medium was administrated with a mechanical injector (Liebel-Flarsheim, USA) at the rate of 0.5 to 1.0 ml/s, which depends on the venography appearance and the operator. The contrast medium was injected for 8 seconds and the total injected volume ranged from 4 to 8 ml. The rotation time for Dyna CT is 6 seconds, and the detector moves at 45° per second. The patients were told to breath as gentle as possible before the Dyna CT and to hold their breath without inspiring or expiring as soon as the injection of contrast medium was initiated. The source power was 90 kVp, and the FOV was 48 cm with a voxel matrix of 512 × 512. To avoid excessive radiation exposure, we did not repeat Dyna CT for the misplaced catheter, the position during repositioning was confirmed by venography by the characteristic pattern of the right adrenal vein.

### Dyna CT interpretation

Dyna CT images were processed and interpreted on a DICOM workstation (OsiriX, Pixmeo, Geneva, Switzerland). Reconstructed axial and coronal images with a thickness of 3 mm were used to evaluate right adrenal vein anatomy. The radiologists adjusted the window level, window center, and magnification as needed. Two radiologists (B.-C.L. and C.-C.C.) with 3 and 12 years of experience independently interpreted the images. A consensus on the anatomical features was reached via discussion between the two interpreters.

**Figure 2:**
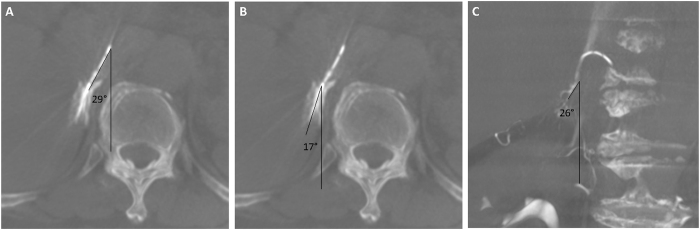
(**a**) A 56-year-old female with primary aldosteronism. Maximal intensity projection (3-mm thickness) of the Dyna CT images showed a well-opacified right adrenal gland. The catheter axial angle was 29°. (**b**) The venous axial angle between the right adrenal vein and the anteroposterior axis of the same patient was 17°. (**c**) Another 59-year-old male with primary aldosteronism. The venous coronal angle between the right adrenal vein and the vertical axis of the patient was 26° in this case.

The anatomical features evaluated were the catheter axial angle, the direction of the right adrenal vein relative to the IVC, the level of the right adrenal vein orifice, and the relationship between the accessory hepatic vein or other accessory veins and the right adrenal vein. The angle between the catheter tip and the anteroposterior axis of the patient on the axial Dyna CT image was defined as the catheter axial angle (Fig. 2A). The direction of the right adrenal vein relative to the IVC was measured as the venous axial and coronal angle. The angle between the right adrenal vein and the anteroposterior axis of the patient on the axial Dyna CT image was defined as the venous axial angle (Fig. 2B). The angle between the right adrenal vein and the vertical axis of the patient on the coronal Dyna CT image was defined as the venous coronal angle (Fig. 2C). Right adrenal veins were evaluated using thick-sliced coronal Dyna CT images to determine the cranial-caudal level relative to vertebral bodies and intervertebral disks. We divided vertebral bodies into superior, middle, and inferior segments.

### Statistical analysis

The Spearman rank correlation coefficient was calculated using MedCalc statistical software (MedCalc version 15.4.0.0, Frank Schoonjans, Mariakerke, Belgium) to evaluate the correlations between the level of the right adrenal vein orifice, the venous axial angle, the venous coronal angle, and body mass index (BMI). For all statistical analyses, *p* < 0.05 was considered statistically significant.

## Results

**Figure 3:**
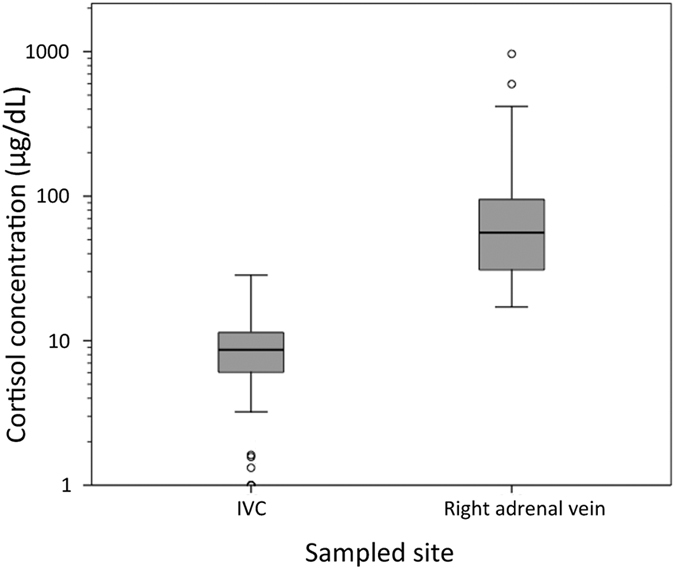
The cortisol level of the sampled blood from inferior vena cava (IVC) and the right adrenal vein. Noted the outlier data were shown in small circles.

A total of 66 patients, 35 males and 31 females with a mean age of 53 years (range 31–73 years), were included in the analysis (Fig. 1). Detailed patient characteristics are summarized in Table 1. The mean cortisol level was 8.79 ± 4.71 μg/dL in IVC, and 101.34 ± 147.40 μg/dL in the right adrenal veins (Fig. 3). There were 6 patients with low (<2 μg/dL) peripheral cortisol level in the AVS result (Supplemental Table S1). AVS revealed unilateral hyperaldosteronism in 48.5% (32/66) of patients. The lateralization results were right side in 27 patients and left side in 5 patients; laparoscopic adrenalectomy was suggested to all these patients (n = 32).

### Location of the right adrenal vein orifice

**Figure 4:**
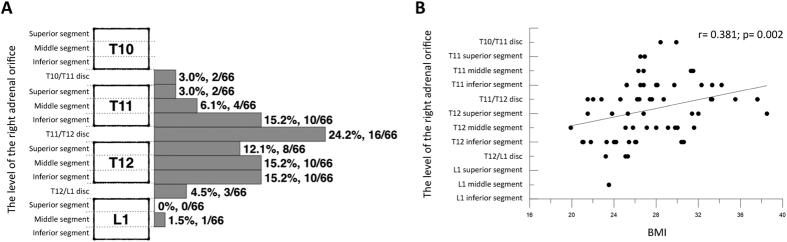
(**a**) The level of the right adrenal vein orifice in relation to adjacent vertebral bodies and discs. Vertebral bodies were divided into three segments. The number and percentage of the right adrenal vein of each segment are showed. (**b**) Correlation between the level of the right adrenal vein orifice and body mass index (BMI). The level of the right adrenal vein orifice was significantly higher in patients with a higher BMI.

Dyna CT location of the orifice of the right adrenal vein is shown in Fig. 4A. The level of the right adrenal vein orifice ranged from the disc of vertebrae T10–T11 to the middle segment of vertebra L1. In 54 (81.8%) of the 66 patients, the level was between the inferior segment of vertebrae T11 and T12. Higher BMI was significantly associated with a higher level of the right adrenal vein orifice (*r* = 0.381, *p* = 0.002) (Fig. 4B).

### Direction of the right adrenal vein

**Figure 5:**
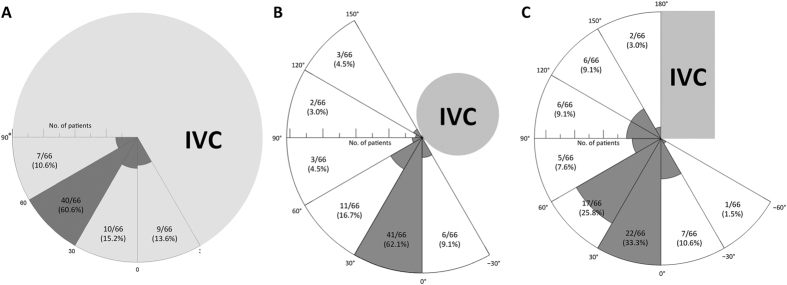
(**a**) Direction of the catheter from the inferior vena cava (IVC) in Dyna CT images, which is represented as the catheter axial angle. (**b**) The venous axial angle, representing the angle between the right adrenal vein and the anteroposterior axis of patients. (**c**) Direction of the right adrenal vein from the IVC in the coronal plane of patients, which is represented as the venous coronal angle.

The catheter axial angle (Fig. 5A) ranged from −19° to 68°. In 86.4% of the patients (57/66), successful catheterization of the right adrenal vein occurred with the catheter axial angle between 0° and 90° (right posterior quadrant). In some difficult cases (13.6%, 9/66), the right adrenal veins were catheterized with the catheter axial angle between 0° to −30°, which required more than 3 attempts for the search of the right adrenal vein. The venous axial angle, representing the angle between the right adrenal vein and the anteroposterior axis of the patients, ranged from −29° to 144°. In 83.3% of patients (55/66), the venous axial angle was between 0° and 90° (right posterior quadrant) (Fig. 5B). The venous coronal angle of the right adrenal vein ranged from −56° to 162° (Fig. 5C). In 14/66 patients (21.2%) the venous coronal angle was between 90° and 162° (cranial direction) and in 52 (78.8%) it was between −56° and 90° (caudal direction). In 8/66 patients (12.1%) the venous coronal angle was below 0°, and in all of them the right adrenal gland was located behind the IVC.

**Figure 6:**
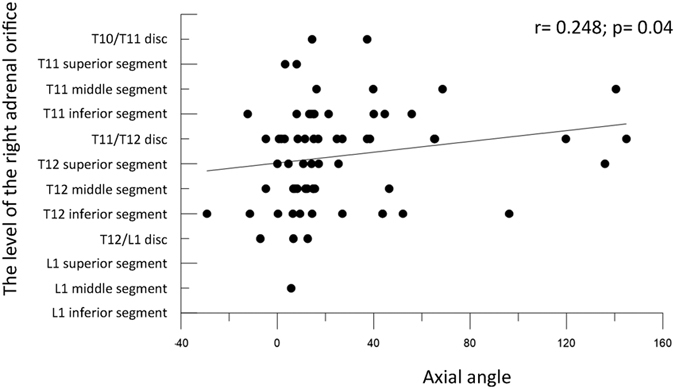
Correlation between the level of the right adrenal vein orifice and the venous axial angle. Higher right adrenal vein orifice level was associated with larger venous axial angle.

There was a statistically significant correlation between the venous axial angle and the level of the right adrenal gland (*r* = 0.248, *p* = 0.04). A higher level of the right adrenal vein orifice was associated with a larger venous axial angle (Fig. 6).

### Accessory vessel of the right adrenal vein

**Figure 7:**
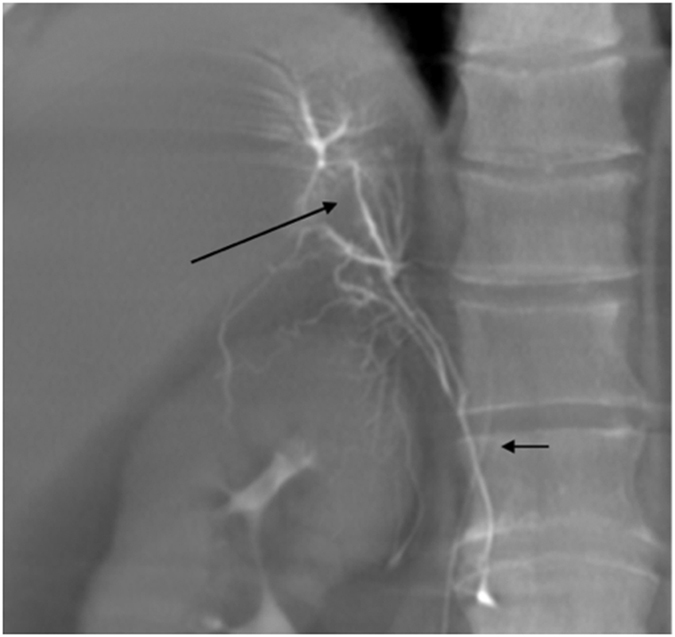
A 48-year-old male with primary hyperaldosteronism. The accessory hepatic vein (long arrow) was opacified during Dyna CT with the catheter tip in the right adrenal vein. The communicating capsular vein of the right adrenal gland was also shown (short arrow).

Right adrenal vein drainage into the accessory hepatic vein was found in 7/66 patients (10.6%), opacification of the right inferior phrenic vein occurred in 16 (24.2%), and communicating capsular veins of the right adrenal glands were opacified in 59 (89.4%) (Fig. 7).

## Discussion

In patients with confirmed PA, AVS is a crucial diagnostic procedure for establishing the subtypes of PA, and to guide further treatments. Despite its importance, AVS has a variable success rate and is a technically challenging procedure. Sampling the right adrenal vein is the most technically challenging part of AVS; therefore, a comprehensive understanding of the anatomy of the right adrenal vein is key for AVS planning, and is even beneficial for future catheter design. Contrast-enhanced CT has been used in several studies to investigate the anatomy of the right adrenal vein^19^,^20^; however, the right adrenal vein is often faintly enhanced after contrast administration, making it difficult to differentiate it from surrounding glandular tissue. The inherent high contrast between the enhanced vascular structure and peripheral tissue is a great advantage of evaluating the course of the right adrenal vein via Dyna CT. Moreover, Dyna CT images generated via a correctly-positioned catheter facilitate a more accurate analysis of the venous structure. In this study, we demonstrated that Dyna CT could delineate the anatomy of the right adrenal vein in detail. Additionally, Dyna CT detected 16 (16.8%) misplaced right AVS catheters in 95 procedures, and 15 (93.8%) of the misplaced catheters were correctly repositioned subsequently.

The orifice of the right adrenal vein ranged from the inferior segment of vertebrae T11 to T12 in 81.8% of patients, which is slightly lower than in previously reported studies using pre-procedural contrast-enhanced CT^19^. This finding is concordant with a report from Daunt *et al*.^21^, who suggested that the pre-procedural CT location of the right adrenal vein orifice is usually 1 cm lower than the actual position. In our study, the patients were instructed to hold their breath at the start of contrast injection and during the Dyna CT scanning, since deep breathing may dislodge the catheter. This is quite different from the conventional contrast-enhanced CT protocol, which usually involves breath holding after inspiration. We believe that the difference between the CT location and actual location can be attributed to this, and knowing this is of great advantage with regard to AVS planning.

We found that the level of the right adrenal vein orifice was related to BMI in patients with PA; higher BMI was significantly associated with a higher level of the right adrenal vein orifice (*r* = 0.381, *p* = 0.002). Iwasaki *et al*.^22^ reported similar findings after retrospectively comparing the location of the venogram during AVS with BMI in 95 PA patients. In addition, we also found a significant correlation between venous axial angle and the level of the right adrenal vein orifice, showing that a higher orifice level was associated with a larger venous axial angle. This unique finding is not mentioned in any previous reports, and may potentially assist in catheterizing the right adrenal vein in challenging cases.

Previous report showed that accessory hepatic vein could be detected in 12.1% of right adrenal venography, whereas no right inferior phrenic veins were detected^23^. In our study, visualization of the accessory hepatic veins (10.6%) was similar to the previous report, whereas detection of the right inferior phrenic (24.2%) vein was relative frequent. This was probably due to the fact that Dyna CT contained 3-dimensional information and did better in delineating the course of the opacified vessels than venography. Moreover, the injected contrast medium was of greater amount in Dyna CT (approximately 4–8 ml) compared to venography by manual injection (usually <3 ml).

All the AVS were performed without ACTH stimulation in this study. In our department, we adopted various ways before and during AVS to minimize the patient stress and to minimize the variability in hormone levels of the sampled bloods. Currently, non-stimulated AVS were used for lateralization of PA in 45% of the world medical centers^24^, since there is no conclusive evidence that ACTH stimulation leads to superior outcomes of AVS so far^25^. In addition, ACTH stimulation during AVS may leads to erroneous lateralization of hyperaldosteronism^26^. The possible mechanism is that ACTH could trigger aldosterone secretion response on both adrenal gland as well as the adenoma with different sensitivity.

Six cases in our study had low (<2 μg/dL) peripheral cortisol level in the AVS result (Supplemental Table S1). Since all these cases were referred from other hospital for AVS, the finding might be related to the dexamethasone suppression test performed elsewhere during adrenal nodules work-up. The lateralization result of AVS in these cases might be devaluated due to the unusually low peripheral cortisol level. However, the selectivity index of these cases were well above 5, which indicated successful AVS. Therefore, their low cortisol levels should had introduced little bias to the derived anatomical information.

AVS is generally a safe procedure, but is not without risks. The reported incidence of major complications in a recent multicenter study ranges from 0.51 to 0.61%^24^. Known complications of AVS include adrenal vein rupture with subsequent hemorrhage, adrenal vein infraction or thrombosis, hypertensive crisis, and adrenal insufficiency, which might be caused by forceful injection of diluted contrast medium during Dyna CT and catheter manipulation. Remission of hyperaldosteronism may occurred months after contrast extravasation or adrenal hemorrhage during adrenal venography^27^,^28^,^29^. In our study, most patients felt no discomfort during Dyna CT, despite a few of them did complained mild flank soreness. One of our cases suffered from adrenal vein rupture with evident contrast extravasation in Dyna CT. The patient had mild back pain on the table and was treated conservatively with analgesics. The result of AVS was bilateral hyperaldosteronism and no adrenal insufficiency was found during follow up.

There are some limitations to our study. First, Dyna CT and the mechanical injection of contrast medium might introduce unknown effect to the AVS result, especially when the adrenal vein was injured after forceful injection during Dyna CT. Second, we only identified the right adrenal vein catheterized by our sampling catheter, and the direction and location of this right adrenal vein might not have been representative if there were two or even three right adrenal veins draining into the IVC separately^14^. However, this is a relatively infrequent condition, reported to occur in 2.5 to 16.8% of right adrenal veins^13^,^30^, and all of the patients enrolled in this current study fulfilled the selectivity criteria of AVS. Therefore, we believe that this possibility did not interfere with our results, based on the successful sampling of the right adrenal vein. Furthermore, the results provide a more practical reference for AVS planning. Third, Dyna CT is inherently more susceptible to artifacts and inferior contrast differentiation than conventional CT^31^. A streak artifact induced by the injection of contrast medium or by the catheter shaft may interfere with interpretation. However, in the current study we excluded images with excessive artifacts and image interpretation was reached by consensus, thus the effects of artifacts were minimized. Fourth, patients with unilateral hyperaldosteronism had uneven distribution of lateralization, in which 27 of them to the right side and 5 to the left. This result might be due to the small sample size of the present study, and probably had little effect on the derived anatomical information.

In conclusion, our study provided a detailed anatomical description of the right adrenal vein in patients with PA during AVS using Dyna CT with a validated catheter tip position. The results were more comprehensive and more accurate than those of previous reports.

## Additional Information

**How to cite this article**: Lee, B.-C. *et al*. Evaluation of right adrenal vein anatomy by Dyna computed tomography in patients with primary aldosteronism. *Sci. Rep.* **6**, 28305; doi: 10.1038/srep28305 (2016).

## Supplementary Material

Supplementary Information

## Figures and Tables

**Table 1:** Patient characteristic of the study population.

Patient characteristics	
Age, year (Mean, range)	53 (31–73)
Gender	
Male, No. (%)	35 (53.0)
Female, No. (%)	31 (47.0)
Height, cm (SD)	163.1 (9.9)
Weight, kg (SD)	73.8 (15.0)
BMI (SD)	27.6 (4.2)
Adrenal nodule	
none, No. (%)	16 (24.2)
right, No. (%)	15 (22.7)
left, No. (%)	22 (33.3)
bilateral, No. (%)	13 (19.7)

BMI: body mass index; SD: standard deviation.
