# Supplementary material for: Evaluation of right adrenal vein anatomy by Dyna computed tomography in patients with primary aldosteronism
Source: Sci Rep. 2016 Jun 23;6:28305. doi: 10.1038/srep28305 (PMC4917856; doi:10.1038/srep28305)
Supplement: Supplementary Information [file srep28305-s1.doc]

**Evaluation of right adrenal vein anatomy by Dyna computed tomography in patients with primary aldosteronism**

Bo-Ching Lee, MD,^1^ Chin-Chen Chang, MD,^1^ Kao-Lang Liu, MD,^1^ Yeun-Chung Chang, MD, PhD,^1^ Vin-Cent Wu, MD, PhD,^2^ Kuo-How Huang, MD, PhD^3^

Departments of Medical Imaging^1^, Internal Medicine^2^ and Urology^3^, National Taiwan University Hospital and National Taiwan University College of Medicine, Taipei, Taiwan

*** Corresponding authors: Chin-Chen Chang, MD**

**Address:** Department of Medical Imaging, National Taiwan University Hospital. No. 7, Chung-Shan South Road, Taipei 100, Taiwan, R.O.C.

**Tel:** 886 2-2312-3456 ext. 62570

**Fax:** 886 2-2322-4552

**E-mail address:** [macotocc@gmail.com](mailto:macotocc@gmail.com)

**Other authors:**

**Bo-Ching Lee, MD**

Address: Department of Medical Imaging, National Taiwan University Hospital. No. 7, Chung-Shan South Road, Taipei 100, Taiwan, R.O.C.

Tel: 886 972653442

Fax: 886 2-2322-4552

E-mail address: [bochinglee@gmail.com](mailto:bochinglee@gmail.com%0D)

[**Kao-Lang Liu, MD**](mailto:bochinglee@gmail.com%0D)

Address: Department of Medical Imaging, National Taiwan University Hospital. No. 7, Chung-Shan South Road, Taipei 100, Taiwan, R.O.C.

Tel: 886 2-2312-3456 ext. 62570

Fax: 886 2-2322-4552

E-mail address: [lkl@ntu.edu.tw](mailto:lkl@ntu.edu.tw)

**Yeun-Chung Chang, MD, PhD**

Address: Department of Medical Imaging, National Taiwan University Hospital. No. 7, Chung-Shan South Road, Taipei 100, Taiwan, R.O.C.

Tel: 886 2-2312-3456 ext. 62570

Fax: 886 2-2322-4552

E-mail address: [ycc5566@ntu.edu.tw](mailto:ycc5566@ntu.edu.tw)

**Vin-Cent Wu, MD, PhD**

Address: Department of Internal Medicine, National Taiwan University Hospital. No. 7, Chung-Shan South Road, Taipei 100, Taiwan, R.O.C.

Tel: 886 2-2312-3456 ext. 62117

Fax: 886 2-2322-4552

E-mail address: [dr.vincentwu@gmail.com](mailto:dr.vincentwu@gmail.com)

**Kuo-How Huang, MD, PhD**

Address: Department of Urology, National Taiwan University Hospital. No. 7, Chung-Shan South Road, Taipei 100, Taiwan, R.O.C.

Tel: 886 2-2312-3456 ext. 62135

Fax: 886 2- 2321-9145

E-mail address: [khhuang123@ntu.edu.tw](mailto:khhuang123@ntu.edu.tw)

Supplemental Table S1

AVS result of the 6 cases with low (< 2 μg/dL) peripheral cortisol level.

| **Case (No.)** |  | **1** | **2** | **3** | **4** | **5** | **6** |
| --- | --- | --- | --- | --- | --- | --- | --- |
| **IVC** | Aldosterone (ng/dL) | 22.2 | 18.5 | 18.4 | 11 | 25.7 | 22.8 |
|  | Cortisol (μg/dL) | 1 | 1 | 1 | 1.5 | 1.3 | 1.6 |
| **Right adrenal vein** | Aldosterone (ng/dL) | 449.2 | 450.6 | 520.7 | 326.9 | 466.8 | 83 |
|  | Cortisol (μg/dL) | 19.6 | 31 | 25.3 | 67.7 | 38 | 45.2 |
| **Left adrenal vein** | Aldosterone (ng/dL) | 156.1 | 321.8 | 209.1 | 31.5 | 486.1 | 219.6 |
|  | Cortisol (μg/dL) | 6.17 | 67.1 | 8.3 | 8.4 | 29 | 8.6 |
